# Subcutaneous tocilizumab in the management of non-infectious uveitis in children: a brief report

**DOI:** 10.1186/s12969-023-00883-y

**Published:** 2023-09-12

**Authors:** Francesca Burlo, Cristina Tumminelli, Serena Pastore, Gabriele Stocco, Debora Curci, Marianna Lucafò, Alberto Tommasini, Andrea Taddio

**Affiliations:** 1 Department of Medicine, Surgery, and Health Sciences, University of Trieste, Trieste, Italy; 2 Institute for Maternal and Child Health “IRCCS Burlo Garofolo”, Trieste, Italy; 3 Department of Life Sciences, University of Trieste, Trieste, Italy; 4 Institute for Maternal and Child Health “IRCCS Burlo Garofolo”, Trieste, Italy

**Keywords:** Uveitis, Inflammation, Biological therapies, Serum, Arthritis, Children, Tocilizumab

## Abstract

**Background:**

Tocilizumab is a humanized monoclonal antibody that acts as an IL-6 receptor antagonist. Intravenous tocilizumab is considered an option for children with anti-TNF refractory juvenile idiopathic arthritis-associated uveitis. In contrast, the potential of subcutaneous drug use with this indication is more controversial. Due to the decreased availability of intravenous tocilizumab during the COVID-19 pandemic, we started using the subcutaneous formulation of the drug in children with anti-TNF refractory uveitis. The study analyzes the serum concentration of tocilizumab and its clinical response in patients with anti-TNF refractory uveitis who started or switched to subcutaneous administration from intravenous use.

**Methods:**

Five patients with non-infectious uveitis were treated with subcutaneous tocilizumab. Ocular inflammation was evaluated on slit lamp examination during clinical control. Serum tocilizumab concentrations were determined by ELISA.

**Results:**

The mean blood concentration of tocilizumab was 61.4 µg/mL (range 2.7–137.0.), with higher values than levels recorded in adult patients with rheumatoid arthritis treated with intravenous tocilizumab. Three patients entered clinical remission. One patient developed a mild relapse and was treated with topical steroids. Only one patient did not respond to therapy. The medication was well tolerated without severe infection or other adverse events.

**Conclusion:**

Our results support a possible role of subcutaneous tocilizumab in anti-TNF refractory uveitis.

**Supplementary Information:**

The online version contains supplementary material available at 10.1186/s12969-023-00883-y.

## Introduction

Pediatric non-infectious uveitis is a rare and sight-threatening condition characterized by inflammation of the uveal components of the eye (retina, choroid, and iris) [1]. Its pathophysiology is not fully understood, and its aetiology is probably multifactorial, with genetic, immune, and environmental factors [2]. Pediatric non-infectious uveitis may be idiopathic in up to 50% of cases, while it is often associated with systemic diseases, including juvenile idiopathic arthritis (JIA) in 40% of cases [1, 3].

Delayed or ineffective uveitis treatments may lead to severe complications, like cataracts, glaucoma, macular oedema, synechiae, and eventually vision loss [1, 4, 5]. A step-by-step approach is recommended to treat uveitis correctly [1]. Topical glucocorticoids can cure mild and transient uveitis, but these medications cannot be used for long due to eye toxicity, with a risk of glaucoma and cataracts. Thus, in refractory cases, starting a systemic immunosuppressive treatment [1, 6] is recommended, usually with methotrexate. In most severe cases, biological treatment is required to control inflammation. Anti-Tumor Necrosis Factor (anti-TNF) agents are recommended for first-line biological treatment, but up to 50% of patients are unresponsive or intolerant [2, 7]. Inhibition may be an effective treatment for the most severe cases of uveitis unresponsive to anti-TNF agents. Interleukin 6 (IL-6) plays a central role in the pathogenesis of autoimmune diseases [2], and its inhibition has been recently shown to be effective in treating the most severe cases of uveitis that are unresponsive even to anti-TNF agents.

Tocilizumab is a humanized monoclonal antibody that acts as an IL-6 receptor antagonist. Even if there is no comparative phase III study, several clinical experiences support its use in patients with uveitis who did not respond to anti-TNF drugs [2, 3, 8–10]. Even the recently published updated recommendations for treating JIA-associated uveitis consider tocilizumab a reasonable choice in cases of Adalimumab failure [11]. According to randomized controlled studies on JIA, intravenous or subcutaneous [12] administration of tocilizumab has similar efficacy, and many authors reported the safety and effectiveness of the subcutaneous administration [11, 13, 14]. However, a recent phase-2 study reported poor effectiveness of the subcutaneous administration for the treatment of patients with anti-TNF refractory JIA-associated uveitis since its primary outcome on treatment response was not completely met [15]. Nonetheless, the new recommendations on JIA-associated uveitis stated that this study demonstrated a clinically relevant treatment response, which can be compared with previous investigations on either adults or children [11]. Therefore, tocilizumab in pediatric patients is almost exclusively used intravenously. The shortage in the supply of intravenous tocilizumab due to its increasing use in positive COVID patients has made necessary the use of alternative treatments, including the administration of the same drug subcutaneously.

We reported five patients with non-infectious uveitis treated with subcutaneous tocilizumab. The primary aim of this study was to evaluate the effectiveness of subcutaneous tocilizumab in refractory JIA-associated uveitis. The secondary purpose was to assess pre-dose serum levels of tocilizumab and correlate them to the clinical response.

## Materials and methods

Our research is a retrospective study on patients treated with subcutaneous tocilizumab for non-infectious uveitis resistant to anti-TNF treatment. The study was conducted at the Rheumatology Service of IRCCS Burlo Garofolo Hospital (Trieste, Italy) according to an IRB-approved protocol. Written consent was obtained from all participants or their guardians.

Slit-lamp biomicroscopy assessed and graded anterior chamber inflammation using the SUN criteria [16]. Dilated indirect ophthalmoscopy was used to evaluate vitreous, retinal, and choroidal involvement. Optical coherence tomography (OCT) was performed in case of posterior pole involvement.

Serum concentrations of tocilizumab were quantified by the Advanced Translational Diagnostic Laboratory of the Institute on the day before the administration, by sandwich ELISA according to the manufacturer instructions (Tocilizumab ELISA Kit ab282910, Abcam, Cambridge, United Kingdom).

This study was approved by the Institutional Board Review at the IRCCS Burlo Garofolo (RC 23/2022).

## Results

We described the history of five patients. They were all female, with a median age of 10 years (average 8–18) at the onset of Tocilizumab treatment. Four were affected by JIA-associated uveitis and one by tubulointerstitial nephritis and uveitis (TINU) syndrome. The median duration of the disease was six years (range 2–17).

Patient 1 was a ten-year-old girl with antinuclear antibodies (ANA)-negative JIA since age one. The articular disease was effectively controlled by methotrexate (15 mg/m^2^ weekly). Nonetheless, when she was four, she presented with a first episode of anterior uveitis of the left eye, followed by recurrent relapses of either ocular or articular disease. After the failure of first- and second-line treatments (Table 1), in November 2021, subcutaneous tocilizumab (162 mg every two weeks) was started, with a consistent improvement. Then, the girl presented with a single relapse of iridocyclitis (visual acuity: 10/10 in both eyes, anterior chamber flare 2 + and vitreous cells 1+, without alterations of the retinal vessels), effectively controlled by topical treatment. At the moment, the disease is still in remission.

**Table 1:** Patients’ characteristics

	Patient number				
	1	2	3	4	5
**Age at onset (yrs)**	4	1	2	13	2
**Associated disease**	JIA	JIA	JIA	TINU	JIA
**ANA**	Negative	Positive	Positive	Positive	Positive
**Duration of disease (yrs)**	6	17	6	2	8
**Previous treatments**	MTX 15 mg/m^2^ weekly MTX + Adalimumab (20 mg every two weeks)	Infliximab (10 mg/kg iv)^*^, Cyclosporine (2,5–5 mg/kg/day) Adalimumab (30–40 mg every two weeks) Tocilizumab (8 mg/kg iv monthly) MTX 15 mg/m^2^ weekly + Tocilizumab (8 mg/kg iv monthly)	MTX 15 mg/m^2^ weekly MTX + Adalimumab (20 mg every two weeks) MTX + Adalimumab (20 mg weekly) MTX + Adalimumab (40 mg every two weeks)	Adalimumab (40 mg every two weeks) Adalimumab (40 mg weekly) MTX (15 mg m^2^) + Adalimumab (40 mg weekly) Adalimumab (40 mg weekly) + Micophenolate (750 mg + 500 mg)	MTX 15 mg/m^2^ MTX + Adalimumab (20 mg every two weeks) Adalimumab (20 mg every two weeks) Adalimumab (40 mg every two weeks) Adalimumab (40 mg weekly)
**Tocilizumab subcutaneous dose** ^*^	162 mg every two weeks (4.2 mg/kg)	162 mg every two weeks (2.2 mg/kg)	162 mg every week (2.7 mg/kg)	162 mg every week (2.7 mg/kg)	162 mg every week (3.5 mg/kg)
**Side effects**	none	none	mild hypertriglyceridemia	none	none
**Duration of therapy with subcutaneous tocilizumab(months)**	10	29	14	14	9
**Remission after sc tocilizumab**	9 months	2 years	16 months	17 months	never reached
**Pre-dose Tocilizumab serum concentrations(µg/mL)**	15.5	2.7	137.0	81.3	70.9
**Other drugs** ^**^	-	-	methotrexate 5 mg per os weekly	mycophenolate 1000 mg/daily	methotrexate 10 mg weekly, prednisone 25 mg daily

JIA: juvenile idiopathic arthritis; TINU: tubulointerstitial nephritis and uveitis syndrome; MTX: methotrexate

* soon suspended because of anaphylactic reaction

** at the time of the tocilizumab hematic levels dosage

Patient 2 was an 18-year-old woman with an ANA-positive JIA associated with bilateral iridocyclitis since age one. Over the years, it effectively controlled the articular disease but failed to control uveitis. Multiple different treatments were tried to handle ocular inflammation (infliximab, cyclosporine, and adalimumab; Table 1), which led to the development of cataracts on the right eye and irido-lenticular synechiae on the left one with visual acuity 8/10 with lenses. On the macular OCT performed at age 10, dystrophy of the retinal fovea and perifoveal intraretinal hypo-reflective areas were found.

Eventually, both the articular and the ocular diseases were well controlled by a combination of methotrexate (15 mg/m^2^) and/or intravenous tocilizumab (8 mg/kg monthly) from age 15 to 17. At the start of the Covid-19 pandemic, tocilizumab was switched to subcutaneous injections (162 mg weekly). Over the following months, the disease was well controlled. After two years, methotrexate was withdrawn, and the tocilizumab dose gradually decreased to one injection every three weeks. To date, the patient remains in remission.

Patient 3 was an eight-year-old girl followed for ANA-positive oligoarticular JIA that began when she was two years old, with right knee joint involvement treated with intra-articular glucocorticoids. Five months later, she presented with the first episode of bilateral iridocyclitis (visual acuity: 10/10 in both eyes, anterior chamber flare 2 + and vitreous cells 2+, no retina involvement) associated with tenosynovitis of the left posterior tibial tendon. After the failure of methotrexate, either alone or associated with adalimumab (Table 1), in October 2021, subcutaneous tocilizumab was started (162 mg every two weeks, then after four months, 162 mg weekly) in association with oral methotrexate (7,5 mg/m^2^ weekly). The girl showed ocular remission and good tolerance to the drug, except for mild hypertriglyceridemia that resolved with diet. At the moment, the disease is still well controlled.

Patient 4 was a 15-year-old girl with TINU syndrome that began when she was 13, with bilateral uveitis (visual acuity: 9/10 left eye, 10/10 right eye, anterior chamber flare 3 + and vitreous cells 3+) and renal tubulopathy (B2- microglobulin 22,290 ng/ml, creatinine 1.01 mg/dL, eGFR 84 ml/min/1.73 m2). A rapid resolution of the tubulopathy was seen after corticosteroid therapy for one month (0.5 mg/kg/day for the first two weeks and 1 mg/kg/day for the other two weeks), while the ocular inflammation was challenging to treat (anterior chamber flare 1 + and vitreous cells 1+, no retina involvement). Adalimumab (40 mg every two weeks) was started, and methotrexate (20 mg weekly) was added two months later for its immunomodulatory effect. Three months later, methotrexate was replaced with mycophenolate (1250 mg/day) to reduce the adalimumab antibodies. Nonetheless, ocular inflammation was never controlled (anterior chamber flare 1 + and vitreous cells 1+, no retina involvement), so adalimumab was stopped, and off-label subcutaneous tocilizumab was started (162 mg weekly), with progressive reduction of inflammation. The girl is still in remission.

Patient 5 was a ten-year-old girl affected by ANA-positive JIA since age two. Initially, she presented just with an articular disease, controlled by methotrexate. Then, since the age of five, she experienced recurrent episodes of iridocyclitis (visual acuity: 10/10 in both eyes, anterior chamber flare 2 + and vitreous cells 1+, no retinal involvement) for which adalimumab (20 mg every two weeks, Table 1) was added with initial benefit. The articular disease was well controlled, and methotrexate was stopped at nine years. However, after one month, she presented another relapse of either articular (proximal interphalangeal arthritis of the fifth finger of the right hand) or ocular (anterior chamber flare 2 + and vitreous cells 1+, no retinal involvement) disease. Adalimumab (40 mg weekly) was switched to subcutaneous tocilizumab (162 mg weekly). After two months, the girl had another relapse of iridocyclitis, so oral methotrexate (15 mg/m^2^) was started (Table 1). Ocular inflammation did not respond to tocilizumab, also when it was administered intravenously for two months (anterior chamber flare 2 + and vitreous cells 1+, no retinal involvement). Then, given the recurrent iridocyclitis and the absence of joint disease, tocilizumab was withdrawn, and intravenous infliximab was started, with a rapid improvement within two weeks from the first infusion (anterior chamber flare 0.5 + and vitreous cells 1+, no retinal involvement).

## Discussion

We started to use subcutaneous tocilizumab at the beginning of the COVID-19 pandemic when intravenous tocilizumab was attempted to treat the hyperinflammatory response associated with the infection. As a result, consuming the intravenous formulation of tocilizumab reduced its availability to treat other kinds of patients, such as children with uveitis. On the one hand, the use of subcutaneous tocilizumab assured continuity of care, while on the other hand, it fulfilled the need to stay at home as much as possible. Moreover, in our experience, self-administered subcutaneous tocilizumab provided an alternative route of administration that was more convenient for both patients and caregivers.

In our experience, subcutaneous tocilizumab was safe and effective in controlling ocular inflammation.

Uveitis is a severe issue in the daily lives of children with this condition. Uveitis affects up to 20% of children with JIA, which is even higher among ANA-positive children [1]. The treatment for uveitis aims to reduce inflammation quickly. In this study, we wanted to report the effectiveness and safety of subcutaneous tocilizumab on uveitis. Regarding side effects, only one patient presented mild hypertriglyceridemia that soon resolved with dietary changes. Therefore, we can state that all our patients did not present significant side effects during the therapy, as already reported [2, 17]. Regarding effectiveness, subcutaneous tocilizumab reduced ocular inflammation in four patients out of five. Another research recently reported on children with JIA-associated uveitis, without significant side effects [14]. Even the last updated recommendations for treating JIA-associated uveitis suggest the use of tocilizumab in cases of adalimumab ineffectiveness, but they do not indicate a preferable route of administration [11]. Some authors speculate that intravenous tocilizumab leads to a more rapid ocular response than subcutaneous administrations [13]. In our experience, patients had rapid improvement with the subcutaneous route. Only patient 2 tried both intravenous and subcutaneous tocilizumab. However, comparing their effectiveness was impossible because many factors may have contributed to the ocular improvement, and her clinical conditions have consistently changed.

Kneepkens et al. have recently focused on serum tocilizumab concentration with clinical response [18]. For example, a concentration higher than 1 µg/mL was reported to be effective in normalizing C-reactive protein levels when administered intravenously [18]. Nonetheless, little is known about the link between drug concentration and clinical response, particularly in pediatric patients or when the drug is administered subcutaneously. We tried to investigate a correlation between pre-dose serum levels of tocilizumab and the effectiveness of the therapy. We found a wide range of serum concentrations. The lowest one (2.7 µg/mL) was detected in patient 2, treated with a low dose of subcutaneous tocilizumab (2.2 mg/kg every two weeks).

Nonetheless, in this case, the serum concentration was quantified during remission of the inflammation, and we do not know the level at the peak of ocular inflammation. Curiously, the highest serum concentration was seen in patient 3 (137,0 µg/mL), who reached an excellent clinical remission and was treated with a low dose of tocilizumab (2.7 mg/kg every two weeks). Interestingly, even patient 5, who did not respond to subcutaneous tocilizumab, presented high serum concentrations (70,9 µg/mL) at a reasonable dosage (3.5 mg/kg weekly). Therefore, from our results, we can state that the drug dosage did not predict clinical response, so we did not meet the secondary outcome of this study. Furthermore, we can state that with the subcutaneous administration, patients reached good hematic levels, as if the drug had been administered intravenously [18].

Nevertheless, we cannot conclude from these results the pharmacokinetic aspects of subcutaneous tocilizumab. However, we can assume that we achieved good suppression of the cytokine IL-6 as reported during intravenous administration [18]. Only one of the patients in our study had received intravenous tocilizumab with a good response; however, it was impossible to compare the different efficacy between the two administration methods. The other patients in our study did not receive intravenous tocilizumab, so we could not compare the efficacy of the two administration methods. This aspect is worth exploring in future studies. Other limitations of this study are its retrospective nature and small patient sample.

## Conclusions

In conclusion, we reported on cases of effective use of subcutaneous tocilizumab to treat pediatric patients with TNF-refractory uveitis. The subcutaneous formulation allowed weekly self-administration with an overall clinical response and a risk-to-benefit profile as good as the intravenous route.

### Electronic supplementary material

Below is the link to the electronic supplementary material.

Supplementary Material 1

## Data Availability

The dataset supporting the conclusions of this article is included within the article.

## Abbreviations

JIA: Juvenile Idiopathic Arthritis
IL-6: Interleukin 6
TINU: Tubulointerstitial nephritis and uveitis
ANA: Antinuclear Antibodies
