# Supplementary material for: Subcutaneous tocilizumab in the management of non-infectious uveitis in children: a brief report
Source: Pediatr Rheumatol Online J. 2023 Sep 12;21:99. doi: 10.1186/s12969-023-00883-y (PMC10496382; doi:10.1186/s12969-023-00883-y)
Supplement: Supplementary file 1 — Supplementary Material 1 [file 12969_2023_883_MOESM1_ESM.doc]

Dr. Francesca Burlo
Department of Medicine, Surgery, and Health Sciences, University of Trieste, Trieste, Italy

16^th^ March 2023

Dear Editor,

On behalf of the authors, I wish to submit an original research article entitled “Subcutaneous Tocilizumab in the management of non-infectious uveitis in children: a brief report” for consideration by Pediatric Rheumatology.

This article is original, it has not been already published elsewhere, and it is not currently under consideration for publication elsewhere.

In this paper, we report on the effectiveness of subcutaneous tocilizumab in treating children affected by anti-TNF refractory uveitis associated with either Juvenile Idiopathic Arthritis or tubulointerstitial nephritis and uveitis (TINU) syndrome. In this study we investigated the clinical effectiveness. Moreover, we measured the pre-dose serum concentration of tocilizumab and tried to investigate possible correlations between this concentration and the clinical response. In our experience, even though subcutaneous tocilizumab was not approved for the treatment of this type of uveitis in children, its off-label use was necessary at the beginning of COVID pandemic when there was a shortage in the supply of intravenous tocilizumab due to its increasing use in positive COVID patients. Surprisingly, the great majority of our patients showed a good clinical response without consistent adverse effects. On one hand, the use of subcutaneous tocilizumab assured a continuity of care, while on the other hand it fulfilled the need to stay at home as much as possible. Moreover, in our experience self-administered subcutaneous tocilizumab provided an alternative route of administration that was more convenient for both patients and caregivers.

We believe that this manuscript is appropriate for publication by Pediatric Rheumatology because it describes a safe and effective use of subcutaneous tocilizumab to treat children with anti-TNF refractory uveitis and, therefore, it may help clinical practice.

We have no conflicts of interest to disclose.

Please address all correspondence concerning this manuscript to me at francesca.burlo@burlo.trieste.it

Thank you for your consideration of this manuscript.

Sincerely,

Dr. Francesca Burlo
